# Combining Feature Selection and Integration—A Neural Model for MT Motion Selectivity

**DOI:** 10.1371/journal.pone.0021254

**Published:** 2011-07-21

**Authors:** Cornelia Beck, Heiko Neumann

**Affiliations:** Institute of Neural Information Processing, University of Ulm, Ulm, Germany; Rutgers University, United States of America

## Abstract

**Background:**

The computation of pattern motion in visual area MT based on motion input from area V1 has been investigated in many experiments and models attempting to replicate the main mechanisms. Two different core conceptual approaches were developed to explain the findings. In integrationist models the key mechanism to achieve pattern selectivity is the nonlinear integration of V1 motion activity. In contrast, selectionist models focus on the motion computation at positions with 2D features.

**Methodology/Principal Findings:**

Recent experiments revealed that neither of the two concepts alone is sufficient to explain all experimental data and that most of the existing models cannot account for the complex behaviour found. MT pattern selectivity changes over time for stimuli like type II plaids from vector average to the direction computed with an intersection of constraint rule or by feature tracking. Also, the spatial arrangement of the stimulus within the receptive field of a MT cell plays a crucial role. We propose a recurrent neural model showing how feature integration and selection can be combined into one common architecture to explain these findings. The key features of the model are the computation of 1D and 2D motion in model area V1 subpopulations that are integrated in model MT cells using feedforward and feedback processing. Our results are also in line with findings concerning the solution of the aperture problem.

**Conclusions/Significance:**

We propose a new neural model for MT pattern computation and motion disambiguation that is based on a combination of feature selection and integration. The model can explain a range of recent neurophysiological findings including temporally dynamic behaviour.

## Introduction

Motion is an important feature of the visual input as it plays a key role for a subject interacting with his or her environment. Whether for social interaction, e.g. a friend waving his hands, or for the recognition of dangerous situations like an enemy approaching quickly, detailed computation of the movement of objects in a scene is a valuable cue. The question is how the visual system generates a proper representation of object motion in order to command decisions. Motion processing in the visual cortex has been a topic of intense investigation for several decades. However, it is still an open question how localized measurements of spatio-temporal changes are integrated and disambiguated, in particular in the case of stimuli provoking non-unique neural responses.

**Figure 1:**
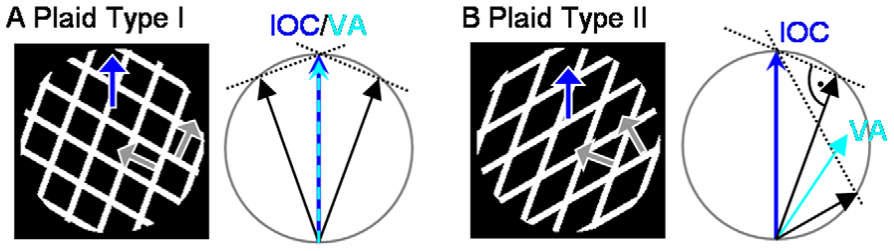
Plaid stimuli. Plaid stimuli are formed by two superimposed gratings consisting of parallel lines that both move in normal flow direction. The stimuli are presented in a circular aperture. **A**) In plaids of type I the direction of the gratings lie on either side of the generated pattern motion. In this case, the vector average of the two motion vectors and the intersections of constraints (IOC) rule will result in (approximately) the same direction. This stimulus was typically used when investigating the pattern response in area MT. **B**) Plaids of type II are characterized by gratings moving in similar direction, i.e. both lying on the same side with regard to the movement that is generated at the 2D crossings. In this case, vector average and the IOC rule will lead to different directions. For this reason, this stimulus provides the possibility to distinguish the computation rule used. Note that a feature signal will lead to the same results as the IOC rule.

**Figure 2:**
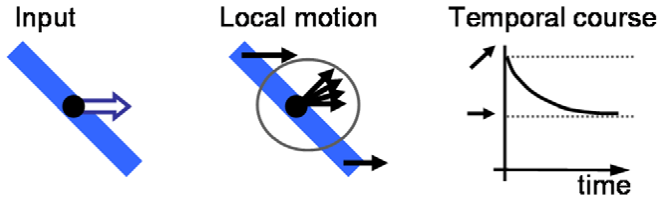
Aperture problem. For an elongated moving contour locally only the normal flow can be estimated. The measured temporal course in macaque area MT [7] indicates for neurons with receptive fields that are spatially aligned along the contour an initial tuning in normal flow direction. This tuning changes over time towards the true motion direction.

Neurophysiological experiments revealed that area MT as part of the dorsal pathway plays a very important role for the computation of motion. The strongest input to this area results from a direct connection with area V1 [1] and the majority of its neurons show motion selective responses [2]. One of the major differences between direction selective V1 and MT cells that has been found is its different response to composed stimuli like a plaid generated by two superimposed gratings oriented in different directions that are both moving orthogonally to their contrasts (Figure 1). As Movshon and colleagues pointed out [3], some MT neurons do not only respond to the components of the plaid, but they are also capable to compute the pattern motion of the presented stimulus (see also [4], [5], [6]). The computation of coherent object motion which may differ from the locally measurable component motion is not only apparent for plaid stimuli. Another example is an elongated contour moving as depicted in Figure 2. Independent of the true motion direction, only the local movement component orthogonal to its contrast can be detected (called “aperture problem”). Recent investigations by Pack and Born revealed that MT neurons do not suffer from the aperture problem, in contrast to the neurons in area V1 [7]. In addition, these authors found that area MT neurons can compute the global motion direction for larger stimuli, e.g., for the barberpole stimulus, again in contrast to responses measured in area V1 [8].

**Figure 3:**
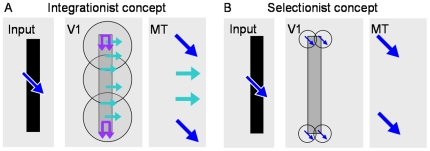
Integrationist and selectionist concept. **A**) A model following the integrationist approach typically has V1 neurons that are component selective, but that do not indicate pattern motion. A nonlinear integration mechanism is then used to compute pattern selective responses in model area MT. The circles in V1 indicate the size of example receptive fields used for integration in model area MT. **B**) Selectionist models are based on a mechanism to find the 2D features in the image as these positions provide 2D motion. Subsequently, Model MT neurons selectively integrate their input to achieve (or inherit) pattern selective responses.

These findings lead to the question how MT achieves the computation of the global pattern velocity, while mainly receiving component selective input from V1. There have been several proposals to explain the generation of pattern selectivity in area MT. Pack and Born [9] suggested a rough distinction of these approaches into two categories depending on the used input features (Figure 3). The so-called “integrationist” models compute the pattern motion by definition based on a nonlinear integration of the V1 input. Simoncelli and Heeger [10], for example, proposed a model that computes the intersection of constraints (IOC) based on the localized activations of V1 cells. Further models have been developed that are driven by this general idea [11], [12], [13], [14]. Unlike the integrationist concept, the “selectionist” models propose to restrict the motion computation to the activity of neurons that respond to 2D features. This has the advantage that the aperture problem does not impede motion processing at these positions since the object-specific localized features already indicate the correct motion direction. Different models have been proposed that follow this idea, for example by [15], [16], [17], [18], [19]. Further approaches exist, amongst other Bayesian approaches [20], [21], models that investigate the interaction between depth and motion information [22]. Another idea is to realize the motion disambiguation process via diffusion mechanisms [23]. Only recently, a model was proposed that emphasizes the role of V1 surround supression for motion integration in area MT, also in the context of plaid stimuli [24].

With respect to the ability of all these models to explain neurophysiological and psychophysical data, we can summarize that these models can explain the pattern selective responses to the commonly used plaid input (compare the review of Pack and Born [9]). However, there remain several challenges which are currently addressed by only few approaches. For example, a number of neurophysiological and psychophysical experiments have shown that the response of MT neurons changes over time from a simple vector average to a direction corresponding to the IOC [25] (see Figure 1) or depends on the contrast of the stimulus [26], [27], [21]. These findings demonstrate that the mechanisms contributing to the pattern computation are part of a dynamic process. The correct tuning takes time to evolve and depends on properties of the stimulus. We suggest that for this process, the interaction of different processing areas is necessary and that the varying behaviour is due to small changes of the input that bias the interaction between the different model areas.

In this paper, we present a neural model that takes advantage of the disparate mechanisms of feature integration and feature selection for motion computation to overcome current model limitations. On the basis of available physiological and behavioral data we show how a neural model of feedforward and feedback interaction between areas V1 and MT including distinct subpopulations of neurons can explain key experimental findings. In particular, the model was probed with stimuli including individual bars of different lengths, type I and type II plaids as well as moving bars in overlay and components displays. Here, we show that the tuning of model MT neurons can replicate challenging experimental findings, namely the disambiguation of responses and the development of pattern selectivity over a time-course of several tens of milliseconds. In particular the question, how plaid II type patterns can be explained is addressed. The specific role of the model subpopulations is demonstrated using lesion experiments. A preliminary version of this paper has been published in abstract form [28].

## Methods

**Figure 4:**
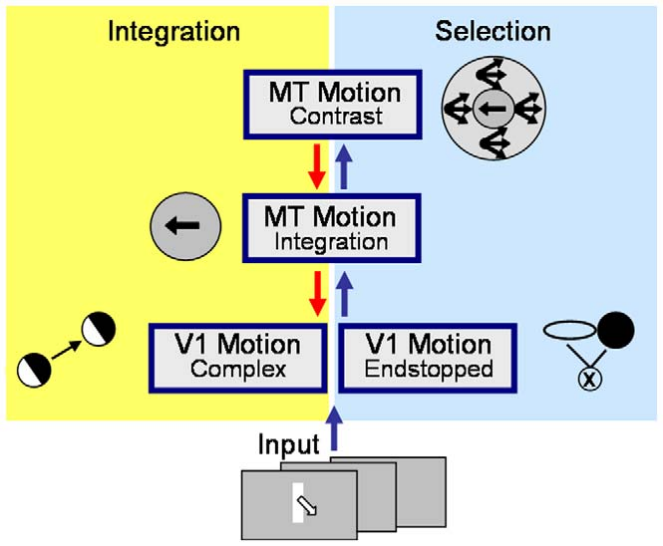
Model overview. Our model includes four neural subpopulations in area V1 and MT. The input enters V1 Complex and V1 Endstopped neurons where motion estimates are computed independently. The computation of endstopped cells takes slightly longer and is for this reason one iteration delayed until the first response is fed forward to area MT. In MT Integration, both inputs are integrated, with a stronger weight and a sharper tuning for the endstopped neurons. Next, the activity is fed forward to MT Contrast. This subpopulation enhances activity of motion surrounded by the opposite motion direction. MT Contrast has feedback connections to MT Integration. The part of the model within the yellow box shows characteristics of integrationist models. Motion computation only in these subpopulations would result in the computation of the vector average. In the blue box, instead, endstopped cells represent a selection process. MT Contrast cells can be assigned to both concepts as they integrate local information, but also contribute to the segementation of image parts moving in different directions.

We propose a neural model that achieves pattern selectivity in area MT based on mechanisms of feature selection and integration. Our approach is inspired by a previous model of motion detection and integration developed by Bayerl and Neumann [29] that simulates areas V1 and MT of the dorsal pathway in visual cortex. In contrast to their proposal, the model areas here include subpopulations of neurons with different properties in both V1 and MT that will be explained in the following subsections (see Figure 4). In a nutshell, the proposed architecture is organized in the following way. (i) Two cell populations in V1 perform the initial motion computation. (ii) Succeedingly, MT neurons integrate the V1 input, followed by (iii) contrast cells (MT/MSTl) responsive to opponent motion directions in the center and surround of the receptive field. The feedforward and feedback connections between the subpopulations allow for an interplay between the different neurons. Each subpopulation contributes to different aspects of motion computation and is as such necessary to achieve the broad range of neurophysiological behaviour.

### Model area V1

In model area V1 the motion of the input stimulus is computed by two subpopulations, namely complex and endstopped cells. The corresponding neurons differ in the way they respond to both the spatial and the temporal components of the input.

#### Complex cells

We simulate complex cells that compute the normal flow of the input [3], [30]. The activity of these neurons is computed with a simple spatio-temporal motion detector for normal flow (compare, e.g., [31]). It will be explained briefly in the following. Initially, the input images are integrated in concentric on-center-off-surround receptive fields. Based on these results, the temporal derivative for two succeeding images is computed as well as the response for different spatial orientations using elongated receptive fields. The responses of these cells, for both the spatial and the temporal domain, are then divided in ON- and OFF-channels to separate positive and negative responses. The direction selective responses of the neurons are achieved by a multiplicative combination of ON- and OFF-channels of both the temporal and the spatial domain. Note that the responses of the spatial domain have to be shifted orthogonally to their contrast to align the elongated receptive fields side by side. The multiplication of the ON- and OFF-channels without the additional temporal factor resembles the computation of a V1 simple cell. After the multiplication, the response of the complex cells is determined by the selection of the maximal responses for the two different contrast polarities. The complex cells will respond most to movement directions orthogonal to the local contrast orientation. Speed selectivity is achieved by filters of increasing spatial size for neurons tuned to higher speeds. As we are currently focusing on the effects of perceived motion direction rather than speed characteristics such a simplistic approach was chosen. To be able to include the comptetitive interaction between the neurons tuned to different speeds, we limited the speeds to the minimum speed tuning that is needed to cover the pixel movements that appear in the images. This led to a speed tuning from 0 pixel to 5 pixel shift with respect to the input image.

#### Endstopped cells

The second subpopulation of model area V1 consists of endstopped cells [32]. The simulation of these cells is based on recent evidence for the existence of V1 endstopped neurons in visual cortex that compute 2D motion [33], [34], [35]. Different approaches which have been suggested include mechanisms of endstopped neurons to compute motion (e.g., [36], [16], [18]. We computed the responses of the endstopped cells for a static image using a recently proposed approach by Weidenbacher and Neumann [37]. Their model consists of two areas V1 and V2 computing the form features including the activity at line ends and crossings. In these model areas and their interactions, key mechanisms at the early stages of shape processing in the temporal pathway are implemented. Visual area V2 is the next stage after V1 in the hierarchy of processing stages along the ventral stream that is assumed to primarily contribute to form processing. Several neurophysiological studies have shown that cells in V2 respond to luminance contrasts, to illusory contours as as well as to moderately complex patterns such as angle stimuli [38], [39], [40]. There is evidence that feedback originating in higher level visual areas such as V2, V4, IT or MT, from cells with bigger receptive fields and more complex feature selectivities can manipulate and shape V1 responses, accounting for contextual or extra-classical receptive field effects [41], [42], [43]. Weidenbacher and Neumann account for these findings by incorporating a recurrent interaction mechanism between model areas V1 and V2 (similar to [44]). In their model, activity in V2 serves as top-down feedback signal to iteratively improve initial feedforward activity in V1. Multiple iterations of feedforward-feedback processing between model areas V1 and V2 lead to more consistent and stable results for the endstopped as well as the other neurons simulated compared to purely feedforward processing schemes. However, the endstopped activity could also be achieved by other mechanisms as proposed in the literature, e.g., lateral connections within V1 or feedback from other areas. Since the model stages of the model of Weidenbacher and Neumann uses essentially the same processing mechanisms, the computation of the responses of the endstopped cells were easily integrated in the feedforward/feedback loop of our model. Just like for the motion information, the results of the form information are improved during the iterations leading to sharper responses. Endstopped cell responses indicate the positions where the local luminance function of the input image has 2D features. The endstopped population receives input from simple and complex cells. Endstopped cells respond to edges or lines that terminate within their receptive field. This includes also corners or junctions where more than one contour ends at the same location. At positions along contours, endstopped cells do not respond. The endstopped cells are modeled by an elongated excitatory subfield and an inhibitory isotropic counterpart which are combined multiplicatively as indicated in Figure 4. The neurons are direction sensitive and are therefore modeled for a set of directions between 0 and 360 degrees. Activities of endstopped cells corresponding to opposite directions are additively combined in order to achieve invariance of contrast direction. These endstopped neurons belong to a processing loop of feedforward and feedback interaction including further neurons in area V1 and V2. The interactions allow to stabilize and increase the responses of the endstopped neurons. Only neurons whose activation exceed a certain threshold are then used for the 2D motion computation. The direction of movement is computed by a temporal integration of the responses of the endstopped cells. Direction selective filters are used to generate responses reflecting the local movement. Like for the complex cells modelled, speed tuning ranges from 0 to 5 pixels shift. An important difference between the motion computation in the two V1 subpopulations is the time that is needed to compute motion signals. While complex cells respond immediately to movement, endstopped cells need one additional iteration to achieve a stable representation of the static 2D features. Subsequently, their activation is sufficient to lead to motion activity.

### Model area MT

In model area MT two different subpopulations are simulated that are mutually interacting, namely MT Integration and MT Contrast, based on findings of Born and Bradley [45]. They differ in their receptive field type and size and in the input they receive.

#### MT Integration

The first subpopulation in the model is called MT Integration and pools the input of the V1 neurons. The mechanism of spatial integration in macaque area MT is one crucial property distinguishing area MT from V1. The receptive field size of the neurons in MT is an order of magnitude larger, compared to the cells that compute V1 activation [1]. The rationale, like in several previous models [10], [29], [11], is that MT cells sample signals over a large variety of directions and over a larger spatial neighbourhood (tuning width approx. +/−90 degree). As such MT cells integrate responses with initial uncertainity and noise component. In our model we use a subsampling of factor five to keep the image size at a reasonable pixel number (a factor of up to ten is indicated in the literature for macaque MT). The input is weighted with a Gaussian kernel in the spatial and the velocity domain. The input of the endstopped cells is weighted more than the input of the complex cells and has a sharper tuning in the velocity domain to take into account that the motion computation of the endstopped cells is more reliable and more precise than the motion computation of the complex cells. This is due to the fact that the endstopped cells signal 2D motion and do not suffer from the aperture problem. The activity of the MT subpopulation is then fed forward to the other neural MT subpopulations, namely MT Contrast.

#### MT Contrast

The MT Contrast subpopulation consists of neurons with an excitatory spatial on-center-off-surround receptive fields organization. The center and the surround are tuned to different motion directions. The cells respond most when the center motion is opponent to the surround motion. For this reason, these neurons support the segregation of objects moving in different directions. This effect can be associated with the selectionist idea as it contributes to the selection of salient positions. At the same time, the integration of motion cues in the center of the on-center-off-surround receptive field contributes to the generation of smooth computed flow, in particular if no opponent movement can be found in the surround. The subpopulation has recurrent connections to MT Integration cells. Neurophysiological evidence for this type of neuron is provided by experiments [46], [47], [48], which showed that the responses in macaque MT can be locally enhanced if the surround contains movement in the opposite direction compared to the center movement. In the current implementation, the delayed response time of the surround in area MT as found in studies by Perge and colleagues is not included explicitly [49]. However, the additional processing step that is included in the model before center-surround neurons in MT are activated would lead inherently to a slightly delayed response of these model neurons.

### Model mechanisms

**Figure 5:**
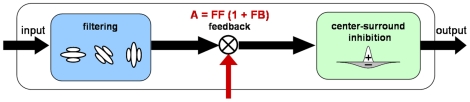
Three-level processing cascade. Each model area is defined by three processing steps. The filtering step differs in terms of the size and the type of receptive field. In general, higher areas in the hierarchy have bigger receptive fields. The receptive field types include concentric and elongated receptive fields as well as concentric on-center-off-surround receptive fields. The following feedback step indicated by the red arrow is a modulatory enhancement of the feedforward input. This means that feedback itself will never create new activity. However, if it matches feedforward input, this activity will be enhanced. The center-surround inhibition is achieved by dividing the activity of each neuron by the overall neural activity at each spatial position. It generates a normalization of the activity within the velocity space.

The implementation of model areas uses rate coding model neurons whose dynamics are described by first-order ordinary differential equations. Within all model areas the same processing mechanisms are applied as depicted in Figure 5. First, the neurons integrate the feedforward input. Second, modulatory feedback of higher areas can enhance the neural activity. Third, in a stage of center-surround interaction the neural activity is normalized with respect to the activity of the neighbourhood of the target cell. This divisive on-center-off-surround competition represents an effect of lateral shunting inhibition where salient signals are enhanced. The following equations give a mathematical description of this generic three step processing:
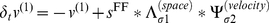
(1)

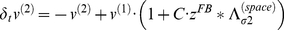
(2)

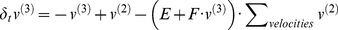
(3)


The terms *ν* ^(1)^, *ν* ^(2)^, and, *ν* ^(3)^ denote the activity within the three stages of the particular model area. The term s^FF^ in (1) denotes the driving input signal, while z^FB^ in (2) is the modulatory feedback signal. The functions Λ and Ψ in (1) and (2) are weighting kernels in the spatial and the velocity domain, respectively, * denotes the convolution operator for filtering operations in space and velocity domain. The constants C and E in (2) and (3) adjust the strength of feedback and lateral subtractive inhibition, respectively. The constant F adjusts the strength of the shunting, or divisive, inhibition. In the results presented here, the steady-state solutions of Eq. (1)–(3) are used to compute the neural activity in order to keep the computational costs in bounds. We have simulated the same model architecture in full dynamics (compare, e.g. [72]) and observed that equilibrated responses did not deviate significantly from those results achieved by steady-state iterations. This lead us to approximate and simplify the computations here. The term “iteration” is used to denote one complete feedforward/feedback processing step. One iteration corresponds to approximately 10–20 ms. We do not take into account here that feedfoward and feedback processing may take a different amount of time. One processing sweep includes the computation of activity in V1, with feedback as computed in the previous iteration in MT, followed by the computation of activity in MT based on the new V1 feedforward input, and the feedback from the activity of MT subpopulations of the previous iteration. The interplay of feedforward and feedback processing is crucial for the model to achieve the expected results. For some model stages, the input activity used in the equations was enhanced by a nonlinear operation. We used the squared activity of the neurons to sharpen the distribution within the neural population. A mathematical description of the equations and the corresponding parameters used at the different model areas can be found in Text S1.

## Results

We tested the proposed neural model with different input stimuli to determine whether the motion computation in model area MT is consistent with neurophysiological and psychophysical results. First, we tested the ability of model MT neurons to solve the aperture problem and to compute pattern motion for plaids of type I. Here, a behaviour similar to the models mainly building on integration mechanisms is shown. Second, the focus was on stimuli that challenge pure integrationist and selectionist models according to our coarse distinction in the Introduction. As one example we utilized plaids of type II where the perceived motion direction changes during presentation time. To clarify the role that the different neural subpopulations of the model play, we conducted several lesion experiments where connections to one of the neural subpopulations were cut successively. Furthermore, we present the results for an experiment where the response of neurons in area MT was tested for small bars moving within the receptive field of one MT neuron, both with overlapping and spatially distinct positions of the bars. This investigation shows how different model functionalities achieve the properties that are indicative of models using feature selection.

With the exception of the results from experiment 4, the results were computed for succeeding input images, i.e. for each iteration one new input image of the sequence was used. In experiment 4, the iterations were based on the same pair of input images (“inplace iterations”) to be able to keep the bars within the same receptive fields. This means that the spatial position of the stimulus did not change during the iterations, only the neural tuning for motion was refined with every iteration. The model parameters remained identical during all experiments.

### Experiment 1: Moving elongated bar

**Figure 6:**
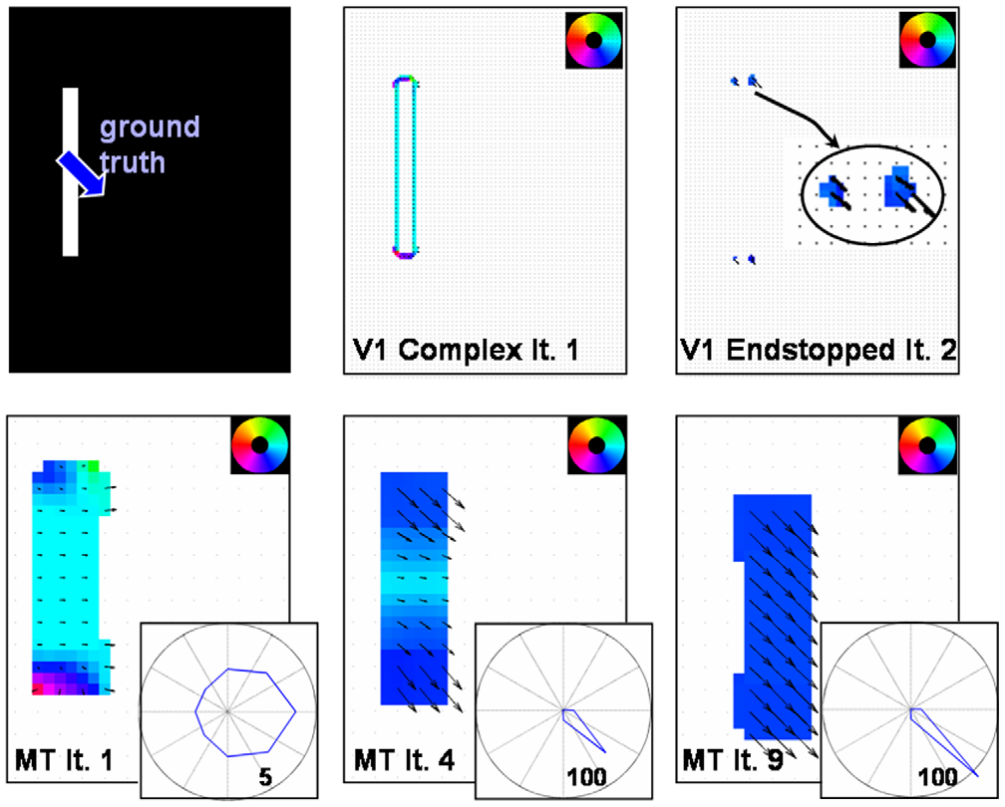
Results of experiment 1. In this figure, the motion tuning within the model subpopulations is depicted. The mean motion direction is indicated by the color code displayed in the upper right corner (e.g., light blue corresponds to rightward motion) and arrows. In some figures, parts are enlarged to allow a more detailed representation (e.g., to show the V1 Endstopped activity in the top row, right). In the model, neurons were tuned to 8 different orientations (Δφ = 45°) and 5 different speeds. **Top row**: A vertically elongated bar is moving to the lower right corner (45°). The mean response of V1 complex cells indicates the normal flow direction from the beginning. V1 Endstopped cells achieve pattern selective responses at iteration 2. The responses of both subpopulations in V1 do not change considerably, for this reason no further results are shown. **Bottom row**: Tuning of MT Integration neurons. After the first iteration, the normal flow dominates at most of the positions. In the bottom right corner a polar plot shows the tuning of all MT neurons active (scaling of radial axis indicated by small numbers in lower right of circles). Initially, the tuning is very coarse with a bias towards the normal flow direction. The disambiguation of motion is visible in the results of iteration 4 and 9 where the true motion is propagated from the corners along the contour until the whole object is moving in a coherent manner.

**Figure 7:**
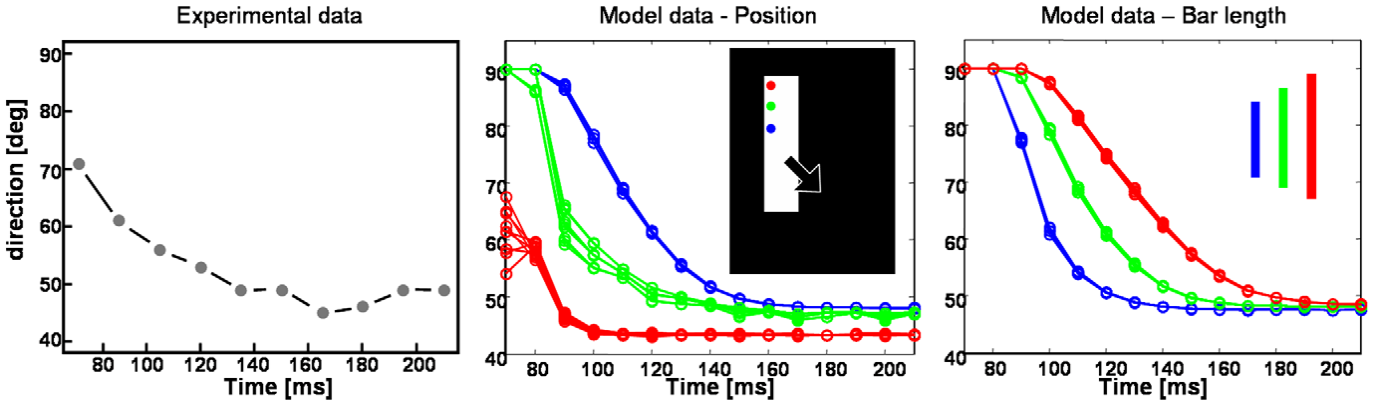
Results of experiment 1 - comparison with neurophysiological data. **Left**: Pack and Born [7] showed in their neurophysiological investigations that the direction tuning of MT neurons located at the elongated contour changes from the normal flow direction (90°) to the correct direction (in this test case 45°; figure adapted and redrawn from [7]). **Center**: Temporal course of our model neurons at three different positions along the bar as indicated by the red, blue, and green dots in the input sketch. The response of the central and the spatially adjacent neurons are shown. The course matches qualitatively with the neurophysiological data. The true motion direction is indicated after approximately 140–150 ms. **Right**: When the bar length increases, the disambiguation process takes longer. This effect observed in experiments of ocular following responses is also replicated by our model. Exemplarily, we show the time course for three different bar lengths indicated by the blue, green, and red bar for neurons located in the center of the bars.

In Figure 6 the results for a vertically aligned bar are depicted that is moving downward to the right (45° diagonal). The input images consist of 170×125 pixels. From the beginning, the response of the complex cells in V1 reflects the normal flow direction of the elongated contour of the bar. In contrast, V1 Endstopped cells respond after a short temporal delay, namely in iteration two, as the computation of its responses needs more time. This has also been found in neurophysiological experiments [33], [50]. As a consequence, the motion computed in area MT initially suffers from the aperture problem. Only when the activity of the endstopped cells starts to feed forward to MT, the disambiguation of the motion to form one coherently moving object begins. Due to the stronger weights of the endstopped cells compared with the normal flow cells, the correct 2D flow propagates with each iteration further along the bar until the whole contour indicates the correct motion. In our model feedback connections to model V1 neural populations are weak, which is in contrast to the model of Bayerl and Neumann [29]. In their model strong feedback caused homologous motion representations in model areas V1 and MT. Particularly, it was predicted that V1 cells solve the aperture problem with a brief delay compared to MT cells. This prediction was in contradiction with experimental findings by Pack et al. [8] who measured responses in normal flow direction along the elongated contours of a barberpole stimulus. In the new model proposed here, we incorporate weak feedback connections from MT to V1. As a consequence, the strength of MT cell influence on V1 computations is reduced such that the tuning of the neurons only slightly changes during the iterations. The solution of the aperture problem in the model proposed here is thus achieved through the interactions between the two different MT subpopulations. We compared this data to the neurophysiological data of macaque area MT [7]. These authors had shown that the mean tuning of MT neurons along the boundary changes from normal flow direction to the correct 2D flow. The temporal evolution of the model responses are qualitatively in line with the temporal course of the experimental data when the iterations are considered as a time scale (1 iteration = 10 ms) and the delay of neural responses is added. One of the predictions of this neural model is that the disambiguation depends on the length of the bar. In Figure 7 (right) the results are shown for three different bar lengths. The time to solve the aperture problem increases with the stimulus length in accordance to recent findings in ocular following experiments [51].

### Experiment 2: Plaid type I

**Figure 8:**
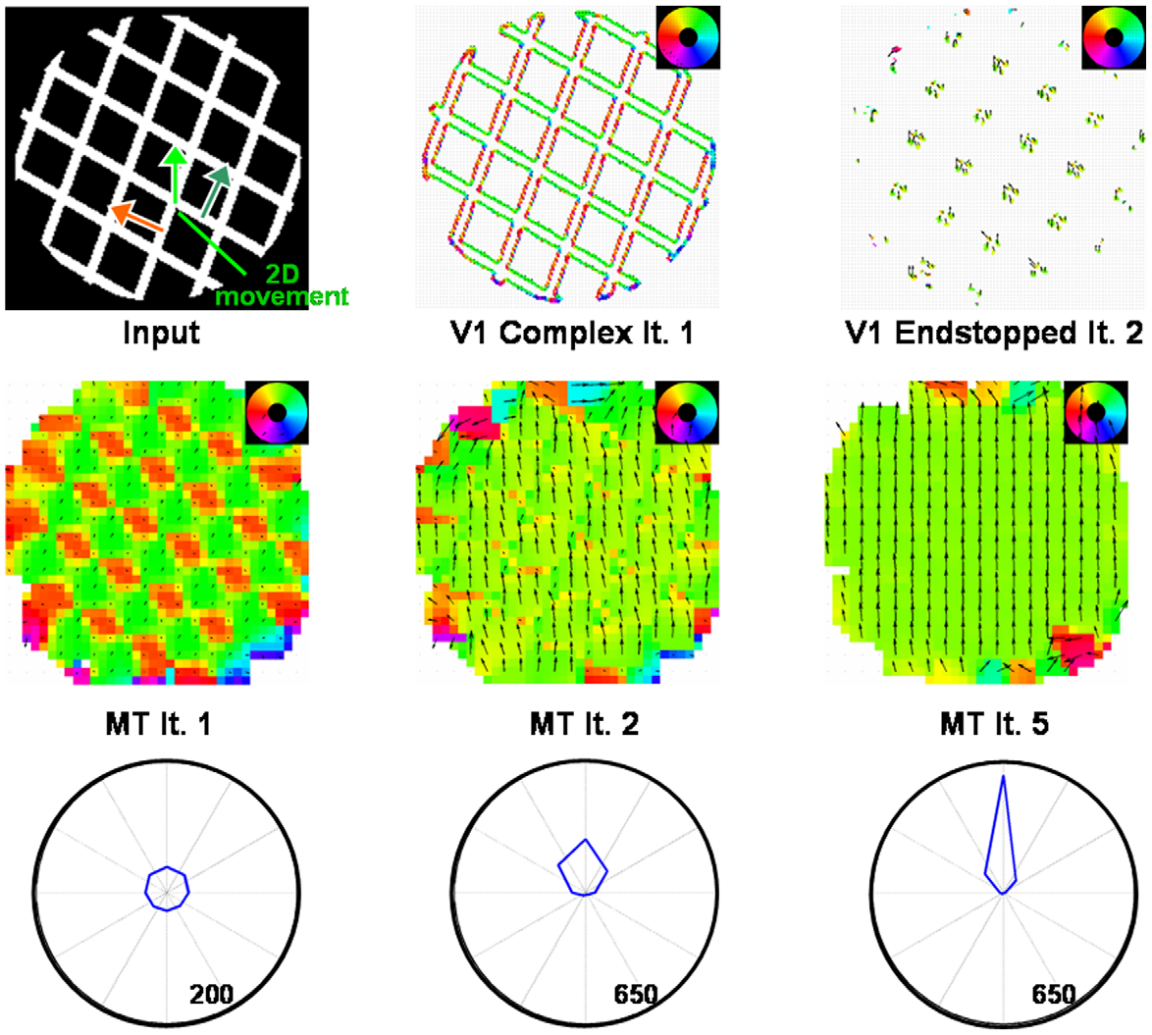
Results of experiment 2. **Top row**: A plaid of type I is used as input, the component and pattern motion are indicated by the coloured arrows. Responses of the complex cells indicate the normal flow direction, V1 Endstopped cells respond from iteration 2 to the motion of the 2D features indicating the pattern direction. **Center row**: Responses of MT Integration neurons. At the beginning, the different motion directions dominate locally indicating component motions. After 5 iterations one coherent motion direction is achieved. **Bottom row**: The polar plots indicate the tuning of MT neurons responding to the plaid pattern for iteration 1, 2, and 5 (note that the scale for the first iteration is smaller than for the other polar plots as indicated by the numbers denoted in the bottom right part of the solid circle). The coarse tuning at the beginning gets quickly sharpened toward the pattern motion direction. The mean velocity corresponds to the pattern motion from the first iteration as both vector average and the 2D motion at the crossings of the gratings indicate the same direction.

When investigating MT motion pattern selectivity, the typical stimulus used is a plaid, two superimposed gratings with similar contrast and spatial frequency that are both moving orthogonally to their contrast boundaries. Alternatively, the plaid can be generated by the overlay of two layers of parallel bars drifting in different directions. Many experiments have shown that the initially perceived motion direction is the coherent pattern motion direction, which corresponds to the movement of the 2D crossings. The perceptual response thus integrates the individual movments of the grating components into one coherent object motion. The combination of component motions corresponds to the vector-average of the inputs. Physiological experiments investigating direction tuning in MT for plaid stimuli also found neurons tuned to the pattern motion. For a plaid of type I the two gratings have a direction that is pointing towards different sides with respect to the pattern motion direction generated (cmp. Figure 1A). As a consequence, for these stimuli the resulting pattern motion can be computed either by taking the vector average or the IOC because they basically indicate the same direction. In Figure 8 the results for an exemplary plaid of type I are depicted (image size 180×180, gratings formed by parallel bars). The V1 complex cells locally compute the normal flow of the two gratings, while the endstopped cells indicate after two iterations the movement at the intersections of the two gratings. Also, at the bar endings 2D movement is detected. Due to the circular shape of the aperture, the direction measured at these positions is not consistent along the aperture. For this reason, these 2D responses cannot generate a strong influence on the whole stimulus. In model MT neurons, the V1 input leads to a combined computation of vector average based on the complex cell input, and an integration of feature tracking like signals based on the input of the endstopped cells. The mechanisms that allow the motion propagation within the context of the aperture problem as presented in the first experiment support here the generation of one coherent pattern. The temporal disambiguation of motion is clearly visible in the polar plot that shows the mean MT directional responses weighted with its activation (Figure 3). While the tuning is very coarse at the beginning, a clear peak emerges after only few iterations.

### Experiment 3: Plaid type II

**Figure 9:**
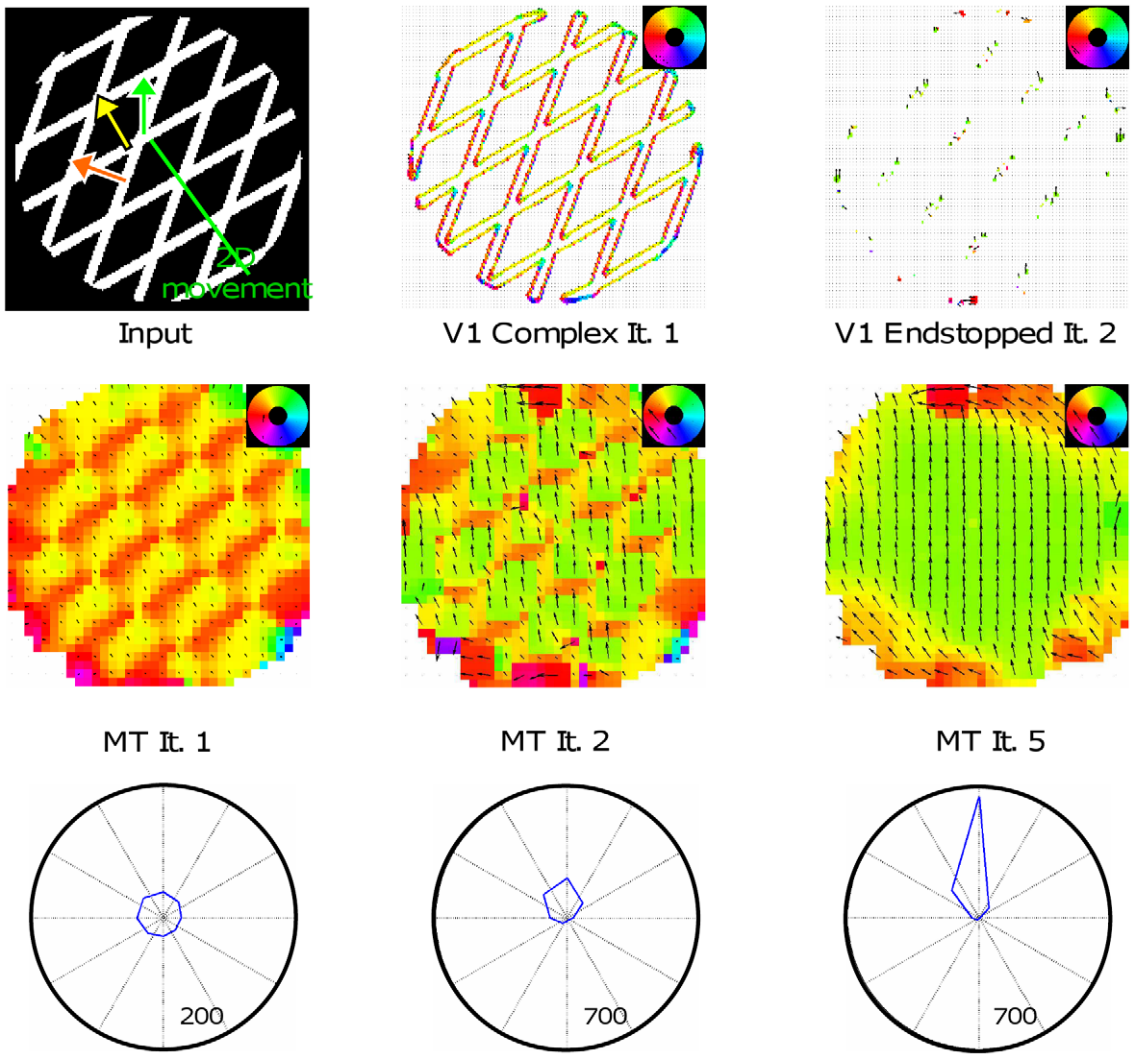
Results of experiment 3. **Top row**: A plaid of type II was used to test the temporal dynamics of the model. The response of V1 complex and V1 Endstopped cells indicate normal flow and pattern motion, respectively, similar to the responses for experiment 2. **Center/bottom row**: After the first iteration, the responses in the direction of the vector average dominate the activity in MT Integration. Once activity of V1 Endstopped cells enter the integration process in MT the overall activity gets shifted towards the pattern direction as the results for iteration 2 show. After five iterations a coherent motion representation is achieved. To sharpen the neural tuning to a similar level reached for experiment 2 some additional iterations are necessary (compare polar plots for iteration 5 and 10).

In plaids of type II the directions of the two gratings lie both on one side with respect to the pattern motion that they generate when moving (cmp. Figure 1B). This entails the possibility to distinguish whether an IOC/feature tracking or a simple vector average of the moving gratings is computed at the stage of MT because they indicate different directions. The results for our model when tested with a plaid of type II are shown in Figure 9. Similar to experiment 2, complex neurons indicate the normal flow direction of the gratings. With a slight delay, endstopped cells indicate the direction in which the 2D features, i.e. the crossings of the gratings, are moving. The integration of these two cell populations in MT now leads to conflicting evidences for motion direction since vector average and feature tracking directions are different. Note that we do not suggest any intermediate stage of representation where the two input regimes are kept separate and subsequently start competing at a neural level. Instead, we argue that the integration of the normal flow responses alone would lead to a computation of the vector average, whereas the integration of the endstopped neurons alone would favor the feature tracking direction which corresponds to the direction indicated by the IOC. Depending on which evidence receives larger evidence the collective response is strongly biased towards either one of the two different possible solutions. The results show that at the beginning the normal flow directions dominate the neuronal tuning as the endstopped cells only respond later. Once the endstopped cells are active, their input starts pushing the tuning of the MT neurons into the direction of the 2D features. After several iterations, MT neurons indicate the pattern motion in a coherent way. Compared with the experiment using the plaid of type I, the disambiguation takes some more iterations as the different motion directions indicated by complex cells and endstopped cells delays the propagation of the 2D motion cues. This behaviour represents a testable model prediction that can be used to verify the model mechanisms by neurophysiological experiments using plaid I and plaid II stimuli.

### Experiment 4: Individual bars in one receptive field

**Figure 10:**
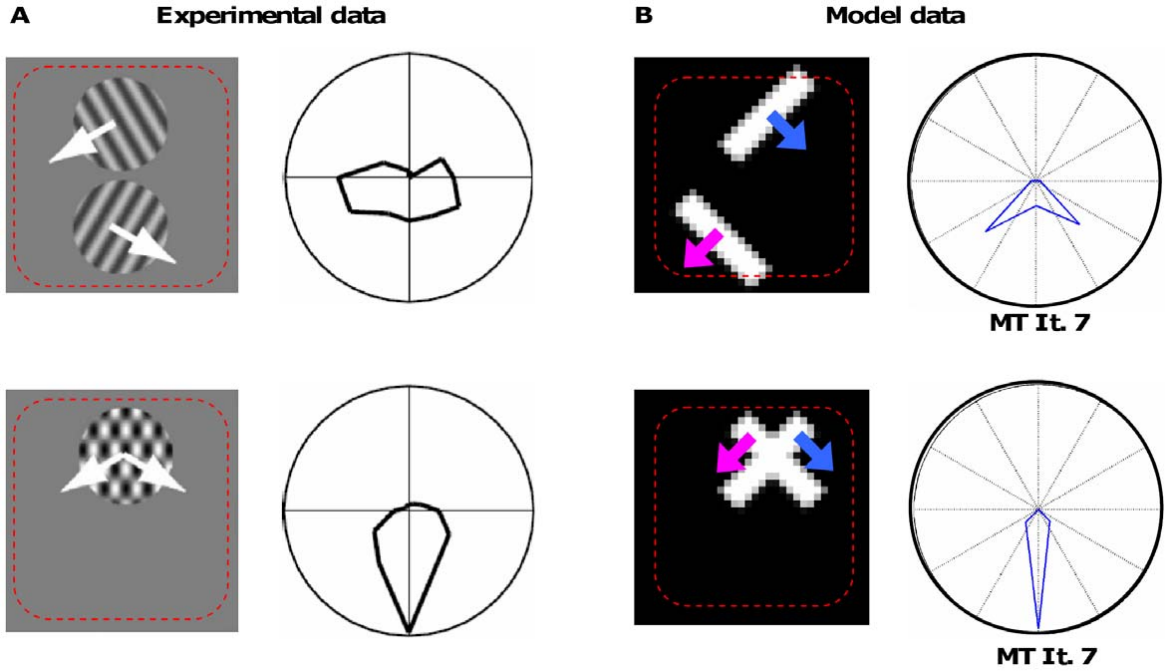
Results of experiment 4. **A**) Experimental results of Majaj et al. [52] (adapted from [56]; note that the direction tuning curves have been rotated 120° clockwise to simplify comparison with our data.). **Left column**: The input stimulus included two moving gratings within one receptive field of a MT neuron. In the first condition, the two gratings were placed at different positions within the receptive field depicted by the red dotted rectangle (top), in the second condition they overlapped (bottom). **Right column**: Response of an MT neuron. When the gratings are not superimposed the response of the neuron is broadly tuned to their component directions (top). For a plaid like stimulus the pattern motion is indicated (bottom). **B**) Adapted version of the experiment to test the model. **Left column**: The tuning of MT neurons was measured for the two cases. In both cases, the size and the movement of the bars (orthogonal to their contrast, orientation +/−45°) are identical. The size and position of the bar was chosen in a way to be mainly within the receptive field of the measured MT neurons as indicted by the red dotted box. **Right column**: The polar plot (radial scale identical for both stimuli) shows that the tuning of MT Integration neurons whose receptive fields includes both bars show a distinct response for the two cases. A bi-lobed tuning appears for the two separate bars that is comparable to the response to the gratings in the experiment of Majaj and colleagues. For the overlapping bars, one clear peak indicating pattern motion is the result.

Another experiment to test the theory of whether MT is simply pooling the input of one cell population as proposed by the integrationist concept is presented in this experiment. We probed MT neurons by stimuli which contain several moving objects at disjoint locations within the receptive field of a cell. If the MT neurons integrated the whole input, then the different object movements would be treated as belonging to one coherent object. We tested our model with a stimulus derived from neurophysiological experiments of Majaj and colleagues [52]. In our experiment, we have two small bars oriented in different directions that are both moving orthogonally to the grating orientation (see Figure 10). The bars only differ in their spatial orientation and the direction of movement, but not in contrast or size. Also, at the position of the intersection the contrast does not differ from the other parts of the bars. For this reason, we assume that the stimulus will not lead to the perception of transparent motion as known for plaids that show differences in contrast or spatial frequency. Nevertheless, we cannot completely rule out the possibility that an effect of transparency may affect the perception in this simplified stimulus. In the first condition, the two bars are located in the upper and lower half of the receptive field of the measured MT neurons without any overlap. To compare effects, in the second condition the two bars are overlapping, forming an “X” whose components are moving in different directions. Note that this stimulus differs from the well known chopstick illusion because of the small size of the bars. Here, the bars are placed in the upper half of one model MT cell receptive field. The results depicted in Figure 10 show that for the first condition, the MT cells with a receptive field center located between the two bars clearly show a tuning with two peaks representing the direction of the two individual bars. For the second condition, the neurons show a different behaviour. After the first iterations, mainly the bar endings show a strong activity indicating their different movement directions, with an additional small activity of the center representing the movement direction of the crossing. After several iterations, the direction tuning of the bar endings shifts continuously towards the movement of the crossing. Finally, the tuning indicates one peak in the direction of the pattern motion formed by the two component bars and their crossing. Due to the combination of two different cell populations a distinct response is achieved for the two experimental conditions. This clearly distinguishes our model from the concept of pure integrationist models which do not have the capability to respond differently to the two cases predicting the same response behaviour, irrespective of the exact stimulus placement within the receptive field.

### Experiment 5: Lesion experiments

To clarify the particular contribution of each of the model subpopulations, we systematically impaired the neural connections in the model. Therefore, the activity of each subpopulation except for MT Integration was silenced successively in several computational experiments. MT Integration itself has not been excluded from the simulations, as it represents the central processing mechanism of the model. Exemplarily, we will focus on the plaid type II stimuli to explain the results.

#### Lesioning V1 Complex Cell input

**Figure 11:**
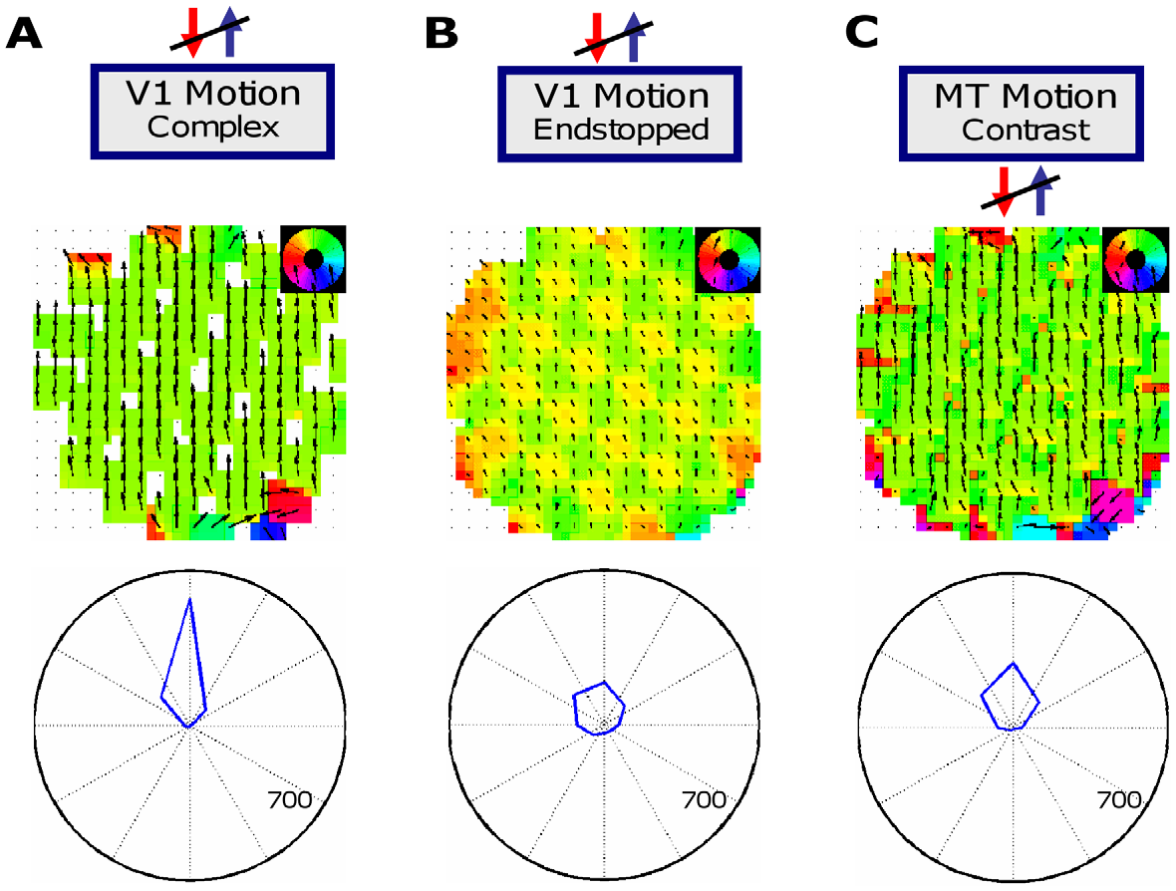
Lesion experiments. In this figure, the results for the plaid of type II input are shown for the model impaired by lesions. Exemplarily, activity in area MT Integration is depicted after 5 iterations. **A**) Cutting the connections from V1 Complex cells leads to MT positions that do not indicate any movements. **B**) When the acitivity from V1 Endstopped Cells is cut off, MT neurons are not able to compute the 2D pattern movement. **C**) Lesioning the connections to MT Contrast also changes the computed pattern in MT Integration. Instead of one coherent motion pattern, the neurons indicate both the normal flow direction and the direction of the 2D crossings.

When the input from V1 Complex Cells to MT is suppressed, the motion computed in model area MT Integration shows two main changes. First, the MT neurons will respond later as the only input comes from the V1 Endstopped neurons which need longer to be activated. Second, not in all MT positions the neurons are activated, there remain void responses where no motion is indicated (Figure 11A). This is a consequence of the missing input along the contours of the bars that form the plaid patterns. It shows that the input of the V1 Complex cells is important to complete the plaid pattern in area MT.

#### Lesioning V1 Endstopped Cell input

The inactivation of input from V1 Endstopped Cells to MT Integration results in an increased tendency of MT motion tuning in the direction of the vector average of the two plaid components. Without endstopped contribution, the movement of the 2D positions formed by the two gratings of the plaid do not influence the MT Integration neurons. For this reason, the model will not show the change of neural activity as presented in Experiment 3 using plaids of type II (Figure 11B). The endstopped neurons are thus the basis in our model to achieve the flexibility to gradually change from the vector average response towards the IOC direction as perceived by humans for plaids of type II.

#### Lesioning MT Contrast Cell input

Also MT Contrast cells play a crucial role in the model. When the connections from MT Integration cells to MT Contrast cells are cut, the plaid motion can no longer be computed correctly. Instead of a smooth global movement direction, MT Integration neurons indicate different directions even after a large number of processing iterations. As a consequence, the stimulus representation remains noisy and incoherent (Figure 11C).

## Discussion

The question how pattern selectivity in visual area MT can be computed has been addressed by a large number of models. Based on neurophysiological findings that supported the computation of an IOC rule or a vector average, the idea of the integrationist concept was seized by several groups. Initial evidence was provided by Movshon and colleagues [3] who showed in both psychophysical and neurophysiological experiments that one coherent movement is perceived for plaids formed of gratings with similar frequency and contrast as indicated by pattern selective neurons in macaque area MT. Furthermore, they performed masking and adaptation experiments whose results further supported the theory of integration of localized movement signals. The results are also in line with data showing that adaptation to one grating with reduced speed biases the overall direction of a succeedingly presented plaid to the non-adapted grating [53]. The idea of the integrationist approach fits also with the investigations of [54] who showed that pattern neurons have a broader tuning than component neurons. Nevertheless, recent research revealed that there is a range of experimental results which cannot be explained by this approach. A number of experiments showed that terminators or 2D features added in a stimulus display can crucially influence the perceived motion direction [26], [55]. The selectionist concept takes the significance of 2D features into account by selecting these positions to compute the pattern motion. However, this concept cannot represent a comprehensive explanation for all the neurophysiological and psychophysical results that have been gathered so far. There is evidence that the process of computing pattern motion shows temporal dynamics that gradually change from a tuning to the vector average to a tuning to the IOC direction. Furthermore, the contrast of the presented stimulus influences the percept [25], [21]. This raises the question whether combinations based on properties of both the integrationist and the selectionist theory could account for the observed data.

We propose here an approach to combine feature integration and feature selection to achieve a broad range of neural behaviour. The key features of our model are

- two neural subpopulations in area V1 that perform distinct computations of motion providing both the normal flow and the flow at 2D features
- a subpopulation in MT that integrates the input of both V1 subpopulations with a more pronounced influence of the 2D (endstopped) features
- feedback connections between MT subpopulations and from MT motion integration stage to V1 subpopulations that allow the propagation and enhancement of salient motion.

In the following subsections we will compare our model with the existing approaches. Furthermore, we will discuss its biological plausibility as well as its potential to account for neurophysiological data.

### Related work

Among the models with a strong emphasis on motion integration, the F-plane model of Simoncelli and Heeger is one of the most influential approaches [10]. MT pattern computation is based on an appropriate weighting of input from area V1 spatio-temporal neurons to compute the IOC. In contrast to our model, no inter-areal feedback connections are used. The model can explain a range of neurophysiological data including data of plaid type I experiments. However, the model does neither show the temporal dynamics that have been observed, e.g., for plaids of type II, nor does it have the capability to segment different small objects as demonstrated with our proposed model.

More recently, Rust and colleagues [11] developed a model to explain MT pattern computation that was derived from neurophysiological data they had measured for plaid stimuli. The two key mechanisms of their model are a strong center-surround inhibition in area V1 followed by a mechanism of motion opponency in area MT. The integration in area MT follows a broad directional tuning curve which is similar to our model in which complex cell responses are integrated by broadly tuned MT cells. The temporal course of responses to plaids, the spatial structure of the normalization pool as well as the influence of the spatial arrangements of the gratings (overlay versus distinct positions) have not been explained by the model. In the approach, a broad directional tuning of MT neurons with respect to the integration of V1 supports the computation of pattern motion which is also reflected in the large tuning comprised by our MT neurons.

A further approach with a strong focus on realistic speed tuning of the simulated neurons was proposed by Perrone and Krauzlis [56]. Their approach differs from other models by modeling V1 and MT neurons that closely replicate the speed tuning curves and the spatio-temporal frequency tuning maps that have been estimated experimentally. Recent results show the replication of the data of Majaj et al. [52]. However, the replication of the dynamics shown in plaids of type II and further experiments has not yet been tested in their model.

Selectionist models could also successfully replicate some of the neurophysiological data. The model of Nowlan and Sejnowski [15] is based on a two stage approach where the motion energy is computed first, followed by a selection of salient 2D features which restrict the position that will enter the final velocity computation. Another model has been proposed by Skottun [17] who used a multiplication (or logical AND-combination) of orientation-tuned filters to compute 2D features. This resembles the use of endstopped cells in our model area V1. However, the computational results achieved here are improved by recurrent processing allowing to arrive at very stable responses. Zetzsche and Barth [16] developed a model where the selection is focused on regions that contain features of multiple contrast orientations, so-called intrinsically 2D structures. While all these models can successfully account for experimental data that measured motion in the direction of the 2D features moving, they do not provide an explanation how changing motion percepts and motion tuning can be generated.

A recent model by Weiss and colleagues [21] uses a Bayesian approach to generate flexible model behaviour. The authors could show the replication of data including both the vector average and the IOC based on an uncertainty value that reflects the local ambiguity of V1 motion estimates. In our model this uncertainty value is implicitly included in the feedforward integration of the two V1 subpopulations in MT. First, the endstopped cells providing unambiguous motion estimates have stronger connections to MT. Second, the integration of complex cells in MT uses a broader directional pooling which results in a reduced activation after the normalization step compared to the sharper input from endstopped cells. Another Bayesian model was proposed by [20] where the focus is on the consistency of information between feedforward and the expected information provided by later recurrent signal. The propagation process of solving the aperture problem looks similar to our result. The computation itself shows different properties due to the different approach of feedforward/lateral activity versus a feedforward/feedback activity here. Detailed plaid results for transparent plaids are shown, but plaids of type II are not considered. Another idea to solve motion disambiguation was prosoped using a luminance-based diffusion mechanism [23]. The model can simulate a range of neurophysiological and psychophysical experiments, including plaids of type II. The focus of their model is on the steering mechanism of motion integration given a luminance-driven representation in the form pathway while we focus on the cascade of motion integration and concentrate on the contribution of the different neural subpopulations found in V1 and MT.

The question how direction selectivity and endstopping interacts in V1 has been investigated in a recent model of Pack, Born and colleagues [57], [24]. Initial motion is detected calculating motion energy by adopting the model of Adelson and Bergen [58]. The output is combined with local inhibitory input from adjacent neurons to generate endstopping properties by center-surround modulation effects in V1 neurons. Subsequently, their activity is integrated in a model MT neuron. The model can replicate detailed neurophysiological response behaviour of MT neurons as measured in Pack and Born [7] including the temporal dynamics from normal flow to the correct flow direction and explain some of the contrast effects on integration properties [27], [7]. Endstopping in their model is generated by temporally delayed pooling of V1 responses in an elongated bi-partite integration field and divisive inhibition of target V1 cell responses by the integrated activity. This resembles computational properties as in our model, since the endstopped responses are generated in our mechanism by gated on/off integration of motion responses (compare Figure 4]. Similar to our model the temporal delay for endstopped neurons is caused by the time it takes to achieve the endstop selectivity. Concerning the interaction of neural areas, the model is based on feedforward integration in one model MT neuron. Their model assumes that the moving bars with their line endings are fully covered by the size of the MT cell receptive field such that no propagation of the 2D motion direction is necessary to resolve the aperture problem. In addition, we predict that the integration of input for type II plaids in the Tsui et al. [24] model is biased towards the vector average. Since their model V1 input responses (with endstopping enabled) do not significantly differ for type I plaid input patterns, their simplified feedforward mechanism is not capable to generate different integrated responses for type I and type II plaid probes. This argument also holds for the challenging display configurations used by Majaj et al. [52]. Again the model proposed by Tsui et al. does not generate distinguishing V1 endstopped responses before integrating them at the stage of their model MT cell. Overall, the focus of their model is on the complex properties that can already be computed in area V1 with a simple integration in area MT. We suggest how the different responses generated by complex and endstopped cells generate different response likelihoods which are disambiguated by the collective integration and feedback signals to account for a disambiguated response at the MT cell level.

In our approach, the interaction between different cortical areas and neural subpopulations with different response properties are crucial to achieve correct result. We claim that this interaction is necssary for the MT motion computation. This link has also been shown in the context of more complex form information in [59], e.g., for stimuli that include spatial occluders. Additional interactions with the form pathway are necessary to compute the correct motion. This has, amongst others, been shown for the barberpole illusion [8], the Chopstick illusion [60] as well as for stimuli including depth-order information [22].

### Biological evidence for V1 model subpopulations

Our model area V1 incorporates complex and endstopped cells which are both connected with model neurons of area MT. This is similar to the model by Loeffler and Orbach [61] who suggested separate streams of complex cell and endstopped responses, respectively, that were kept separate to compute Fourier and non-Fourier motion. Unlike their proposal, which explicitly argues against endstopping, we utilize mechanisms selective for 1D and 2D input features. Complex cell responses are computed by a simple spatio-temporal motion detector with elongated receptive fields for the computation of orientation selective responses. The resulting motion tuning shows strongest responses to the normal flow with ambiguous responses at 2D features. We chose this sort of motion detector as it represents a very simple way to model basic properties of V1 spatio-temporal filters as measured by [3], [30]. However, the stage could also be replaced by a more refined model of V1 computing properties.

The second subpopulation that we simulate is based on the response of endstopped neurons. The existence of endstopped cells responding to 2D static features has already been shown by Hubel and Wiesel [32]. Only few years ago, a study by Pack and colleagues [33] demonstrated that also 2D motion signals are computed by endstopped neurons in macaque area V1. Furthermore, Tinsley et al. [35] as well as Guo et al. [34] measured the selectivity of V1 neurons to pattern motion. Until now, it has not been unequivocally demonstrated that these neurons are indeed projecting to neurons in area MT (e.g., [62]). However, based on the origin of the measured neurons in layer 4B of area V1 which contains a large population of neurons projecting to area MT, the contribution of endstopped cells to MT pattern computation is very likely (see [9] for a detailed discussion). Concerning the shape of the supressive receptive field, [63] showed that length suppression for these celles is stronger than side suppresion as realized in our model endstopped neurons. In the current implementation of the model, for simplicity only the two extremes of purely complex and endstopped cells are modeled to show how these subpopulations may contribute to the motion computation. In neurohphysiological findings, it has been shown that these two classes of cells have large overlaps. This model simplification is one reason for the fact that our simulation results generated by the model are sharper than the measured neurophysiological data. Furthermore, our current experiments do not contain additional noise inputs.

### Replication of neurophysiological data

A large number of neurophysiological experiments has clarified and constrained the computation of pattern motion in area MT. With the model proposed here, we focus a) on the temporal course of responses and b) on the different mechanisms, namely vector average and IOC/feature tracking, that seem to be applied in MT as shown by various experiments [26], [25], [21]. In the first experiment we tested the ability of our model to perform a crucial property observed in macaque area MT neurons, namely the solution of the aperture problem. The results (Figure 6 and 7) confirm that our model can propagate the 2D movement measured at the line endings along the contour and that it shows a similar time course compared to the neurophysiological data. The computation is mainly achieved during the feedforward/feedback processing of MT Integration and MT Contrast. Spatial propagation of the correct motion direction detected at the corners is necessary to achieve the correct direction for the whole elongated object as its extent is much larger than the size of the corresponding MT receptive fields. For object segmentation based on motion, the bigger receptive fields of MT can be combined with the more detailed form information of area V2 as has been shown in further computational experiments [59], [64]. The longer time needed for disambiguation (Figure 7) that appears with increasing bar length is consistent with data measuring the ocular following responses for tilted bars of different lengths moving horizontally [65]. The reduced response strength of MT neurons in the first iteration is not in line with the neurophysiological experiments by [7] where the spike rate per ms in area MT cells is stable over time for the single neurons measured. However, in our model only the mean response rate is represented and a discretized time scale is used. Further experiments need to be done to see whether this effect can be reduced when the simulation based on iterations of the steady-state equations is replaced by the stepwise solution of the model differential equations, following the experiments of [72]. At present, the difference of the activity level arises as the feedback only interacts after the first iteration.The question whether the computation of the motion disambiguation is also reflected in the V1 responses is still unclear. There is some recent evidence that V1 responses change their tuning [66] contradicting the previous findings [3], [30], [8]. In the current model version, we show that a changing tuning of V1 complex cells is not necessary to achieve the motion disambiguation. Concerning area MT, studies revealed that approximately 50% of MT neurons show center surround characteristic [46], [67]. These findings are the reason why we incorporated two different types of MT cells our model, namely integration and contrast cells. We assume that the interplay of the different neural response characteristics leads to the final neural interpretation of data.

The constrast neurons simulated in the model could also be found in area MSTl. There is evidence from neurophysiological experiments that this cell type appears both in area MT [45] and area MSTl [68]. Area MSTl is known to contribute to the detection of small moving objects, for this reason a contribution of these neurons to the computation of patterns that include strong 2D features like plaids seems possible.

The propagation of salient motion features is also relevant for the computation of pattern motion when presenting plaids. In experiment 2 and 3, we showed the results for plaids of type I and II (see Figure 8 and 9). For the type I stimulus, both the vector average indicated by the integrated normal flow responses as well as the feature tracking/IOC signal provided by the endstopped cells point into approximately the same direction. For this reason, the computation of the coherent plaid motion is achieved after few iterations. For the results using a plaid of type II, two differences are noticeable. First, the initial MT responses clearly indicate movement in vector average direction, which then turns into the IOC direction once the endstopped neurons get active. Second, due to the different directions indicated by the two V1 subpopulations, the disambiguation process takes longer than for the plaid of type I. The observation that pattern selectivity only emerges slightly after component selectivity has also been found in neurophysiological and psychophysical investigations. The temporal course of MT cell tuning for plaids and gratings shows an earlier response of component selective neurons while pattern selective neurons show a brief time-lag in their response characteristic [6]. Masson and Castet [69] showed that the ocular following responses of humans for a plaid stimulus have a delay of about 20 ms until the pattern direction is pursued, confirmed by the investigations of Born and colleagues [50]. In line with that data, Yo and Wilson [25] found that for a short presentation time, plaids of type II appear to be moving in the direction of the vector average. Our model gives a plausible explanation for these effects, as its dynamics depend on the activation of two subpopulations in V1 that have different time courses.

The recent experimental results of Majaj and collaborators [52] represent a further challenge for models simulating MT pattern selectivity. Unlike, e.g., the model of [56] and our approach, many of the existing approaches do not take into account spatially distributed locations and can therefore not account for this data. For the simplified stimulus that we used – the gratings were reduced to single bars – the tuning responses of model area MT neurons look similar to the responses measured. This behaviour is achieved due to the different responses of the endstopped cells that contribute to the MT motion computation. It allows the switch from a bi-lobed tuning (indicative of component selectivity) to a clear peak (indicative of pattern selectivity) when a plaid is presented. The psychophysical experiments by Mingolla et al. [55] addressed the question how the visual system integrates boundary movements to form a coherent percept by utilizing separated apertures each containing distinct stimulus components. Their results argue in favor of a hybrid mechanism that combines vector average and feature motion integration. Our model supports this view by suggesting area MT motion computation that is flexible to compute partial motion from translational, rotational or more complex pattern movements.

The perception of plaids has also been studied for much longer presentation times. Hupé and Rubin [70] showed that if one observes the plaid stimuli for 20 seconds and longer the percept switches from the pattern configuration to a bi-stable percept where pattern and the component configuration of two transparent gratings alternate. In our model, the two different cell populations simulated in area V1 would represent a good basis to represent bi-stability as they basically reflect the two different percepts that are competing. At the level of MT, our subpopulation of pattern selective neurons would have to be extended by a component in our model which was currently not needed but which would allow to adapt the excitation after a brief period of persistent input stimulation. For example, this could be achieved at the level of input integration by incorporating transmitter habituation (e.g., [71]). The introduction of such a fatigue mechanism allows to take into account bi-stability as generated by mechanisms with mutually competing response selectivities (e.g. opposite motion directions).

### Predictions and outlook

The presented model makes predictions that could be tested by neurophysiological experiments to gain further knowledge about motion processing in area MT. First, our model predicts that due to the two different cell populations in area V1 contributing to MT activity, a sort of competition between endstopped and complex cells occurs for type II plaids. For this reason, the temporal course should be delayed if compared to the response of type I plaids. Second, endstopped activity in V1 is generated in a feedforward-feedback loop with V2 form activity in our model. Thus, cooling of area V2 should reduce endstopped selectivity in neurophysiological experiments. We would further predict that, as a consequence, the reduced endstopped activity will lead to a change of activity in MT for type II plaids. The complex cell input would dominate the MT activity leading to a bias towards the vector average response of the moving gratings. In fact, this converges to the investigation of the detailed mechanisms of temporal V1 responses, as studied by Tsui and colleagues [24] and the model proposed here. Our model operates and takes into account a larger scale of neural computational mechanisms involved to achieve different response properties and selectivities. A layout of a generalized model framework that demonstrates receptive field computation at a population level and the (delayed) response normalization effects by integrating pooled activation has been recently proposed by Bouecke et al. [72].

In future experiments, we will take a closer look at experimental results where the switch between vector average and IOC direction is due to the strength of contrast. It has been shown that endstopped cells are contrast selective [73], [74]. Reduced responses of the endstopped cells in our model would reduce its influence during the integration in area MT. This would be a possible explanation for the perceived motion in vector average direction when a thin rhombus is displayed at low contrast [21]. In this case, the weak endstopped responses would hardly contribute to the MT input. As a consequence, the “integrationist” part - the complex cell input - would bias the overall computation considerably, leading to a tuning towards the vector average. The strength of contrast has also been linked to changing behaviour of MT models in the contrast of solving motion disambiguaty. Either antagonistic or integrative properties have been found and modeled [75]. Further investigating these properties in our model could be a key to simulate other neurophyiosological findings.

### Conclusion

We suggest a new neural model for MT pattern computation and motion disambiguation that can account for a number of recent neurophysiological findings. This model proposes a combination of feature selection and integration for motion computation in area MT. Thus, we are able to replicate seemingly conflicting experimental data in one common framework that achieves temporally dynamic behaviour including responses to the vector average and to the IOC/feature tracking at different time steps.

## Supporting Information

Text S1 **Mathematical description of the model.**
(DOC)
