# Supplementary material for: Combining Feature Selection and Integration—A Neural Model for MT Motion Selectivity
Source: PLoS One. 2011 Jul 21;6(7):e21254. doi: 10.1371/journal.pone.0021254 (PMC3140976; doi:10.1371/journal.pone.0021254)
Supplement: Text S1 — Mathematical description of the model. (DOC) [file pone.0021254.s001.doc]

# Text S1. Mathematical description of the model

The following subsections give a detailed mathematical description of the model equations used. As mentioned in the model section, the steady-state responses of the generic processing mechanisms were utilized to compute the neural activation. In the following equations, the terms *ν*^(1)^, *ν*^(2)^, and, *ν*^(3)^ denote the activity within the three stages of the model area. Weighting kernels in the spatial and the velocity domain are denoted as Λ and Ψ, respectively, * denotes the convolution operator, **x** the spatial position. φ denotes the direction of motion, *s* the corresponding speed. The constants C*_ModelArea_*, G, F and H adjust the strengths of feedback, multiplicative enhancement and lateral inhibition.

## V1 Motion

The initial motion estimation in V1 can be distinguished in neurons with activity ***v***_1D_ and ***v***_2D_ that provide 1D (V1 Complex) and 2D motion detection (V1 Endstopped).

### V1 Complex

Initial motion estimation *I_1D_* is computed by a spatio-temporal motion detector using speeds ranging from 1 to 5 pixels in 8 different directions. The detector is based on two succeeding input images convolved in the spatial domain with a Laplacian of Gaussian filter (Equation 1) followed by a temporal derivation by computing their difference *dLoG_t_* at time *t* (Equation 2). In addition, the activity for the orientation of the local contours orthogonal to the motion direction is computed by elongated receptive fields(*oLoG_t_*) representing subfield for the computation of complex cells (Equation 3). All these activities are separated in the absolute value of positive and negative responses ([*dLoG*]^+^,[-*dLoG*]^+^, etc.). The direction of motion is detected by selectively multiplying the responses of the elongated subfields and the temporal derivatives, depending on the contrast polarity. Before the multiplication, the subfields have to be shifted spatially (by speed/2 pixel along and against the direction of motion indicated as *Left* and *Right* in Equation 4) to get the appropriate spatial alignment. As an example, rightward motion (φ=0°) is both indicated by a) a high responses of the positive responses of o*LoG_t_* on the left side, of the negative responses of *oLoG_t_* on the right side and of positive responses of *dLoG_t_* in the center and b) a high responses of negative activity of *oLoG_t_* on the left side, of positive responses of *oLoG_t_* on the right side and of negative responses of *dLoG_t_* in the center (Equation 4). Speed selectivity is achieved by using receptive fields of different size (cmp. Equation 1). Zero motion is not represented explicitly. The motion detection was restricted to the regions where zero crossings had been detected in *LoG_t_*.

$LoG_{t}=Input_{t}*\Lambda_{LOG(\sigma=speed/2)}$ (1)

$dLoG_{t}=LoG_{t}-LoG_{t-1}$ (2)

$oLoG_{t}= LoG_{t}*\Lambda_{\sigma\_x=speed/2;\sigma\_y=speed}^{^{elongated}}$ (3)

(4)

The responses of this motion detector is the activation of the V1 Complex cells (Equation 5). In the second processing step, feedback ***v***_MT_ from model area MT enhances the neural activity cells (Equation 6). Third, the activity is normalized with respect to the activation of the neurons tuned to different speeds and directions at the same postion (Equation 7).

$v_{V1\_1D}^{(1)}=I_{1D}$ (5)

$v_{V1\_1D}^{(2)}=v_{V1\_1D}^{(1)}\cdot\left(1+C_{V1\_1D}\cdot v_{MT}^{(3)}*\Lambda_{\sigma1\_V1}^{\left(\mathbf{x}\right)}\right)$ (6)

(7)

### V1 Endstopped

Initial motion estimation ***v***_2D_ is like for ***v***_1D_ computed for a speed range from 1 to 5 pixels in 8 directions. The responses of endstopped cells of four succeeding image frames are used to generate a temporally blurred response. A detailed description of the mathematical equations used to compute the response of the endstopped cells can be found in [37]. Only the activities exceeding a threshold θ are used for the motion computation. Motion direction is achieved using direction selective filters formed by the combinations of two elongated gaussian filters. In the following, the same processing steps are applied like in model area V1 Complex (Equations 8-10).

$v_{V1\_2D}^{(1)}=I_{2D}$ (8)

$v_{V1\_2D}^{(2)}=v_{V1\_2D}^{(1)}\cdot\left(1+C_{V1\_2D}\cdot\sum_{s} (v_{MT}^{(3)}*\Lambda_{\sigma1\_V1}^{\left(\mathbf{x}\right)})\right)$ (9)

(10)

To improve speed tuning of the cells, an additional inhibitory competition was included after step 2 (Equation 9). Neurons tuned to speed = 1 were divisivly inhibited by $(B+D\cdot\sum_{s>1} v_{V1\_2D}^{(2)})$. This inhibition was necessary to account for the problem when measuring a minimum spatial shift of one pixel with receptive fields that comprise several neurons. This led to a positive bias for the neurons tuned to the smallest speeds which is corrected using this inhibition. To control the activity, both constant B and E have a rather high value (5 and 10, respectively).

## MT Motion

In model area MT the input of the V1 subpopulations is integrated and further processed. In MT integration, which receives the input from V1 neurons, the spatial resolution is reduced by factor 5 to take into account the coarser spatial representation observed in visual area MT.

### MT Integration

In the first processing step of model area MT the input of the two V1 subpopulations is integrated using a gaussian filter in the spatial and the velocity domain (Equation 11), with a stronger blur of speed and direction for the complex cells and a strong amplification of the input from the endstopped neurons (F=100). This is necessary to allow a quick change of directional tuning for the MT cells once the reliable information of the endstopped neurons is available. The feedback from MT Contrast is slightly blured with respect to speed and direction (Equation 12). Finally, the squared activity is normalized (Equation 13). The neural responses are sharpened by the quadratical function used in these equations. However, we do not apply it in every processing stage to avoid instabilities.

(11)

(12)

(13)

### MT Contrast

In this model area, the neural response is increased if motion in the opponent direction is found in the surround. After spatial integration of the squared MT input (Equation 14), the responses of the center and the surround of the receptive field are computed. The center activities are an integration of the activities of the neurons tuned to similar directions weighted with a gaussian filter applied in the spatial domain. The surround activity is based on the activities of neurons tuned to the opponent direction, again weighted with a spatial gaussian filter. The center response modulatory enhanced by the response of the surround (Equation 15) is then used as feedback to the activity computed in step 1. In the third processing step the activities are normalized (Equation 16). Here, the normalization is in the direction domain restricted to neurons with similar direction tuning to reduce the strong competition for neurons tuned to different directions.

$v_{MTC}^{(1)}=E\cdot\left(v_{MT}^{(3)}\right)^{2}*\Lambda_{\sigma1\_MTC}^{\left(\mathbf{x}\right)}$ (14)

$v_{MTC}^{(2)}=v_{MTC}^{(1)}\cdot\left(1+\left(v_{MTCon}^{(1)}\right)\cdot\left(1+C_{MTC}\cdot v_{MTCOff}^{(1)}\right)\right)$ (15)

$v_{MTC}^{(3)}=\left(v_{MTC}^{(2)}\right)/\left(H+J\cdot\left(\sum_{\text{s}} v_{MTC}^{(2)}\right)\right)$ (16)

In Eq. (15) the activations $v_{MTCon}^{(1)}$ and $v_{MTCoff}^{(1)}$are determined by the following convolutions

$v_{MTCon}^{(1)}=v_{MTC}^{(1)}*\Lambda_{\sigma\_MTC\_cen}^{\left(\mathbf{x}\right)}$ and

$v_{MTCoff}^{(1)}=v_{MTC}^{(1)}*\Lambda_{\sigma\_MTC\_surr}^{\left(\mathbf{x}\right)}$.

| $C_{V1\_1D}$ | $C_{V1\_2D}$ | $C_{MT\_1}$ | $C_{MT\_2}$ | $C_{MTC}$ |
| --- | --- | --- | --- | --- |
| 0.5 | 1 | 50 | 100 | 100 |
| $\sigma1\_V1$ |  | $\sigma1\_MT\text{s}$ | $\sigma2\_MT$ |  |
| 1 | 0.5 | 2.25 | 5 | 1.25 |
| $\sigma2\_MTs$ | $\sigma3\_MT$ |  | $\sigma3\_MT\text{s}$ | $\sigma1\_MTC$ |
| 1 | 1.5 | 0.7 | 0.7 | 3 |
| $\sigma\_MTC\_cen$ | $\sigma\_MTC\_surr$ | A | B | D |
| 1 | 3 | 0.01 | 5 | 10 |
| E | F | G | H | J |
| 10 | 100 | 0.1 | 0.8 | 0.01 |

Model parameters used. The parameters were identical for all experiments presented.
